# Current State of Robotics in Interventional Radiology

**DOI:** 10.1007/s00270-023-03421-1

**Published:** 2023-03-31

**Authors:** Ghazal Najafi, Kornelia Kreiser, Mohamed E. M. K. Abdelaziz, Mohamad S. Hamady

**Affiliations:** 1 Department of Surgery and Cancer, Faculty of Medicine, Imperial College London, London, SW7 2AZ UK; 2 Department of Neuroradiology, Rehabilitations - und Universitätskliniken Ulm, 89081 Ulm, Germany; 3 The Hamlyn Centre, Imperial College London, London, SW7 2AZ UK; 4 Department of Electrical and Electronic Engineering, Faculty of Engineering, Imperial College London, London, SW7 2AZ UK

**Keywords:** Robot, Endovascular, Interventional radiology, Robotic systems, Image guided robotics

## Abstract

As a relatively new specialty with a minimally invasive nature, the field of interventional radiology is rapidly growing. Although the application of robotic systems in this field shows great promise, such as with increased precision, accuracy, and safety, as well as reduced radiation dose and potential for teleoperated procedures, the progression of these technologies has been slow. This is partly due to the complex equipment with complicated setup procedures, the disruption to theatre flow, the high costs, as well as some device limitations, such as lack of haptic feedback. To further assess these robotic technologies, more evidence of their performance and cost-effectiveness is needed before their widespread adoption within the field. In this review, we summarise the current progress of robotic systems that have been investigated for use in vascular and non-vascular interventions.

## Introduction

Interventional radiology (IR) is one of the most innovative and creative disciplines, with a steady stream of developments in imaging techniques, catheters and devices as well as treatment procedures. Nevertheless, IR has been lagging behind other specialities when it comes to robotics. The DaVinci robot was first used for laparoscopic cholecystectomy in Belgium in 1997 [1] and has been widely used since 1999, especially in visceral surgery, urology and ophthalmology. Orthopaedic surgeons have been implanting robotic-assisted endoprostheses since 2000 [2]. Much later, angiographic robots were invented initially for use in cardiology, and subsequently transitioned into the field of IR [3–5].

In the field of IR, the robotic catheterisation systems aim to improve 1) the precision and safety of the operation and 2) the access and comfort of the patient, while 3) minimising operator skill variability and 4) reducing radiation exposure to both patients and clinicians. In addition, given the teleoperated nature of these systems, their benefits can be made accessible to patients in rural and underserved populations. Similarly, the application of robotics to non-vascular IR procedures provides the opportunity to improve the precision of percutaneous procedures with enhanced adherence to the predefined target path. In this paper, we will explore the current robotic advancements in endovascular and non-vascular IR procedures.

## Robotic Endovascular Procedures

Over the past two decades, several commercial and research platforms have been developed to assist interventionalists in peripheral vascular (PVI), neurovascular (NVI) and percutaneous coronary interventions (PCI) [3, 6–13]. A summary of the key characteristics of these robotic systems is listed in Table 1 and a summary of the clinical studies undertaken using these robots is found in Table 2.

**Table 1:** Summary of the main characteristics of robotic systems for endovascular IR

Robotic system	Institute	Regulatory status	Compatibility with off-the-shelf equipment	DOF of robot	Procedures
Sensei	Hansen Medical Inc., USA	FDA	No	3 DOF	Cardiac mapping, ablations
Magellan	Hansen Medical Inc., USA	FDA	No	7 DOF	PVI (aortic stent grafting, FEVAR, UAE)
CorPath GRX	Corindus, Siemens Healthineers, Waltham, MA, USA	FDA, CE mark	Yes	5 DOF	PCI, PVI, NVI
R-One	Robocath, Rouen, France	CE mark	Yes	N/A	PCI
Niobe ES	Stereotaxis Inc., USA	FDA	No	3 DOF	Cardiac mapping and ablation
Amigo	Catheter Precision Inc.m USA	FDA	Yes	3 DOF	Cardiac mapping, ablations

*DOF* degrees of freedom; *PVI* peripheral vascular intervention; *PCI* percutaneous coronary intervention

**Table 2:** Summary of the clinical studies of endovascular robotic systems

Study	Robotic system	Type of study (# subjects)	Aim	Key findings
Riga et al. [4]	Sensei	Case report (1)	5.9-cm infrarenal aneurysm repair	Completed EVAR No post-op complications Stent-graft well-positioned at discharge and 3-month post-op
Lumsden et al. [41]	Sensei	Case report (1)	Stenting of an anastomotic pulmonary artery in-stent stenosis	Re-stented the stenosed site No procedural complications
Carrell et al. [16]	Sensei	Case report (1)	Repair of kinked renal bridging stent 8 months following a branched endovascular repair of a type III thoracoabdominal aortic aneurysm	Realigned the kink with an additional stent Restored renal perfusion 6-month post-op patient required intermittent renal dialysis
Bismuth et al. [42]	Sensei	Prospective, single arm (15)	Overcome navigation difficulties in iliofemoral arteries in PAD	100% lesions accessed 19/20 limbs treated with balloon angioplasty No peri-procedural complications Navigation time and radiation dose differed between interventionalists
Riga et al. [18]	Magellan	Case report (1)	7.3-cm juxtarenal aneurysm repair	Completed FEVAR No post-op complications CT at discharge and 4-month post-op revealed vessel patency with no evidence of endoleak
Rolls et al. [19]	Magellan	Prospective, single arm (5)	Bilateral UAE	100% technical success Median fluoroscopy time: 11 min No peri-procedural complications
Cochennec et al. [43]	Magellan	Prospective case series (15)	Target vessel cannulation in complex endovascular aortic procedures	81% cannulated within 15 min 19% converted to conventional method Median wire cannulation time: 263 s No robotic-related intraoperative complications
Lu et al. [44]	Magellan	Case report (1)	Embolisation of ascending aortic pseudonaeurysm	Embolised and occluded the aneurysm No procedural complications
Perera et al. [22]	Magellan	Non-RCT (11)	Cerebral embolisation in robotic-assisted vs manual TEVAR	Total 6 HITS with robotic vs 38 with manual procedures (*p* = 0.018)
Owji et al. [45]	Magellan	Case report (1)	IVC filter retrieval	Retrieved IVC filter No procedural complications
Schwein et al. [46]	Magellan	Case report (1)	Type II Endoleak embolisation	Completed embolisation without complications
Giudice et al. [47]	Magellan	Prospective, case series (21)	Comparing the performance of Magellan V 1.0 and V 1.1 in UFE	UFE completed in 57.1% with Magellan V 1.0 vs 100% with Magellan V 1.1 (*p* = 0.01) Fluoroscopy time (*p* = 0.03) and radiation dose (*p* = 0.04) were lower with V 1.1
Jones et al. [20]	Magellan	Prospective case series (13)	Carotid artery stenting	100% technical success defined as navigation to arch and stabilisation in the CCA No procedural complications
Caputo et al. [26]	CorPath GRX	Prospective case series (5)	Renal artery stenting	100% technical success defined as completion without conversion to manual operation or deployment of an additional stent Achieved < 30% residual stenosis in all No adverse outcomes
Smitson et al. [77]	CorPath GRX	Prospective, multicentre, open-label, non-randomised, single arm (40)	PCI for obstructive coronary artery disease (> 70% stenosis)	Final TIMI 3 flow and < 30% residual stenosis without any major adverse outcomes was achieved in 97.5% Technical success (not needing to convert o manual) occurred in 90.0%
Al Nooryani et al. [25]	CorPath GRX	Case report (1)	PCI for lesion of proximal to mid LAD artery	Final TIMI 3 flow and no evidence of residual stenosis Stented LAD artery No intra-operative complications Successful use of RoR function
Swaminathan et al. [78]	CorPath GRX	Case report (2)	Trans-radial diagnostic angiography	Successfully manoeuvred catheter to visualise coronary vasculature in both patients
Hirai et al. [79]	CorPath GRX	Case report (1)	PCI of LMCA in patient with history of ALCAPA	Successful stent positioning and deployment Peri-operative complications
Mendes Pereira et al. [27]	CorPath GRX	Case report (1)	Coiling of basilar artery aneurysm	Successful stent-assisted coiling of basilar artery No perioperative complications
Piotin et al. [28]	CorPath GRX	Prospective, multicentre single arm (113)	Coil and/or stent-assisted coiling embolisation of at least one unruptured cerebral aneurysm	Embolisation success rate of 94.7% Five subjects underwent conversion to manual operation Median procedure time: 114.3 ± 43.5 min Median fluoroscopy time: 52.1 ± 27.3 min
Robocath (Rouen, France) [31]	R-One	Prospective, non-randomised, single arm (62)	PCI of coronary lesions	Technical success rate of > 95% with a 100% clinical success rate No device related complications post-op Average reduction of 84.5% in radiation dose to the physician 3/62 required total manual conversion
Da Costa et al. [80]	Niobe ES vs Niobe II	Retrospective case series (184)	Quantifying exposure parameters in AFib ablation using Niobe ES versus Niobe II	Lower procedure time by 30% (*p* < 0.0001) Reduced fluoroscopy duration by 30% (*p* = 0.001)
Yuan et al. [81]	Niobe ES	Retrospective case control (214)	Comparing outcomes in AFib ablation using Niobe ES vs manual technique	Median fluoroscopy of 10.4 min for Niobe ES versus 16.3 min for manual (*p* < 0.001) No significant difference in total procedure time At 3.5-year post-ablation, AFib-free survival was significantly better with Niobe ES
Kataria et al. [82]	Niobe ES	Retrospective case control (336)	Comparing long-term outcomes in RFA of paroxysmal AFib using Niobe ES versus manual technique	Freedom from repeat ablation was 70.9% in Niobe ES versus 69.5% in manual Majority of repeat procedures took place in first year in both groups No differences in complication rates between the groups
Luo et al. [83]	Niobe ES	Retrospective case control (110)	Assessment of steerable sheath compared to fixed-curve sheath in AFib ablation guided by Niobe ES	Steerable sheath allowed reduced procedure time and radiofrequency time (*p* < 0.001) No procedural complications
Khan et al. [35]	Amigo RCS	Prospective, multicentre, non-randomised, single arm (181)	Evaluating Amigo for navigation and positioning of mapping catheter	Eight sites were mapped with a success rate of 96% No major procedural complications One minor adverse event of atrial tachycardia which was likely Amigo-related
Datino et al. [36]	Amigo RCS	Prospective, single centre, non-randomised, two-arm (100)	Comparing the safety and feasibility of Amigo versus manual technique in arrhythmia ablation	Procedure success rate, procedure time, and RF delivery time was similar between the two groups Amigo group had an average reduction of 68 ± 16% operator radiation exposure No procedural complications
Lopez et al. [37]	Amigo RCS	Prospective, multicentric, single arm (60)	RFA of cavo-tricuspid isthmus (CTI) in typical atrial flutter	98% successful, stable, bidirectional CTI block One conversion to manual No complications related to Amigo
Wutzler et al. [38]	Amigo RCS	Prospective, dual-centre, non-randomised (119)	Comparing Amigo versus manual technique in ablation for paroxysmal AFib	Successful ablation in all patients for both groups No difference in procedure time, total energy delivered, and total fluoroscopy time Mean operator fluoroscopy exposure in Amigo was 13.4 ± 6.1 min compared to 23.9 ± 5.4 min for manual (*p* < 0.001) No procedural complications

*EVAR* endovascular aneurysm repair; *PAD* peripheral arterial disease; *FEVAR* fenestrated endovascular aortic repair; *CT* Computerised tomography; *UAE* uterine artery embolisation; *QoL* Quality of Life; *TEVAR* thoracic endovascular aortic repair; *RCT* randomised control trial; *HITS* high intensity transient signals; *IVC* inferior vena cava; *UFE* uterine fibroid embolisation; *CCA* common carotid artery; *PCI* percutaneous coronary intervention; *TIMI* thrombolysis in myocardial infarction; *LAD* left anterior descending; *RoR* rotate on retract; *LMCA* Left main coronary artery; *ALCAPA* anomalous left coronary artery from the pulmonary artery; *AFib* atrial fibrillation; *PV* pulmonary valve; *RFA* radiofrequency ablation; *RF* radiofrequency

### Sensei and Magellan

Sensei (Hansen Medical, Mountain View, CA, USA) was one of the first commercially available robotic system that obtained the US Food and Drug Administration (FDA) approval in 2007 to be used in cardiac mapping and ablative procedures [14]. This system enabled robotic control of a steerable guide catheter remotely using 3 degrees of freedom (DOF) joystick [15, 16]. Although Sensei provided better catheter stability in comparison with manual procedures and was successfully used for cardiac ablation and endovascular aneurysm repairs, mechanical issues related to the system profile and applicability were reported using this system [17]. The next generation of the robotic platform from Hansen Medical was the Magellan robotic system, which received its FDA 510(k) clearance back in 2012, and allowed interventional radiologists to remotely control the shape and movement of the distal co-axial tip of 6Fr, 9Fr, and 10Fr robotic catheters and the robotic manipulation of standard off-the-shelf guidewires. The robot is able to control the movements of 0.035″ and 0.018″ wires, and the operator is able to advance, retract, rotate in 360 degrees and park the wire by using buttons in the robot control station. The pioneering robotic system has shown its efficacy and safety in several peripheral arterial interventions such as aortic stent grafting, fenestrated endovascular aneurysm repair (FEVAR) and embolisation techniques [18, 19]. Through several individual cases and in small, selected case series, this system has demonstrated certain benefits, such as reduced vessel wall damage and embolic events with better control of vessel centreline navigation, improved stability while navigating tortuous anatomy, enhanced cannulation success of target vessels, improved movement economy and reduced radiation doses to operators [4, 18–24]. However, the main limitations of the Magellan were the high installation and running costs, as well as the inability to integrate all therapeutic devices.

### CorPath GRX

In contrast to the discontinued Magellan, the FDA-approved and CE-marked CorPath GRX (Corindus, Siemens Healthineers, Waltham, MA, USA) facilitates the control of third-party guiding catheters, guidewires, and therapeutic balloon/stent catheters. The GRX platform, the successor of the CorPath 200, includes additional advanced procedural automation movements (FDA- cleared 2018 and 2020) such as rotate on retract (RoR) [25] wiggle, spin, dotter and constant speed. The main applications of the GRX systems are for use in PCIs and NVIs. Nonetheless, the applications of this system in other procedures have also been explored, such as for percutaneous renal stent implantation in five patients [26]. In 2020, the CorPath GRX robotic system was used in a stent-assisted coiling procedure of a basilar artery aneurysm [27]. A prospective, multicentre single-arm trial, recently presented in a congress [28], has evaluated the procedural technical success and the incidence of intra-and peri-procedural complications using the CorPath GRX in 113 patients with at least one unruptured cerebral aneurysm requiring endovascular coil and/or stent-assisted coiling embolisation [28, 29]. Robot-assisted embolisation success rate without the need to convert to manual operation was 94.7%. In order to complete the procedure, five subjects underwent conversion to manual operation [28]. The results of this trial are yet to be published.

Whereas the Magellan robotic platform uses dedicated robotic catheters of 6Fr, 9Fr, and 10Fr, the CorPath GRX system uses commercially available 5-7Fr guiding catheters, which is partially responsible for making the CorPath GRX system more cost-effective in comparison. Using the CorPath GRX, the operator is able to use the joystick in the control station to advance and retract off-the-shelf catheters. The platform is currently able to accommodate 0.014″ wires. Although the initial cost of acquisition of the Magellan system, estimated at around $600 K, is similar to the GRX system with figures ranging between $480-650 K, the cost of each disposable Magellan robotic catheter is $1500 compared to $400–750 for the single-use cassette of the GRX system [11, 30]. While there is no direct comparative study between CorPath GRX and Magellan systems, it is the view of the author who has had experience with both devices (MH), that the technical abilities of CorPath GRX, such as navigation, stability and applicability across a range of anatomical variations are likely inferior to its predecessor. This is related mainly to the inherent feature of CorPath GRX which uses standard off-the-shelf catheters with no added mechanical features.

### R-One

Another robotic platform which offers a similar solution to the GRX is the R-One robotic PCI system (Robocath, Rouen, France) that received CE marking in 2019. The R-One allows interventionalists to manipulate off-the-shelf guidewires and stent/balloon catheters (excluding a guiding catheter). The R-One was used in the R-Evolution clinical trial in a non-randomised, prospective single-arm clinical trial [31]. Sixty-two patients requiring stent implantation were enrolled across six European centres. The findings of this clinical trial identified that the technical success rate for this system is > 95% with a 100% clinical success rate. No device-related complications were observed post-procedure, and the robotic assistance allowed an average of 84.5% reduction in radiation dose to the physician. Total manual conversion was required in three patients [31].

### Niobe ES

Niobe ES (Stereotaxis Inc., MO, USA) is a commercially available magnetically driven robotic platform that implements magnetic fields to navigate and relocate custom-made magnetic catheters in 3 DOF. The magnetic catheter is made up of soft material to avoid excessive contact force and reduces the risk of cardiac perforation [17]. The main drawbacks of Niobe are related to its need for costume designed catheters, relatively long set-up time of roughly 30 min, and the need for a large space to place the device [14]. In 2020, Stereotaxis introduced Genesis, an updated version of the Niobe system, which incorporates a novel design with a reduced robot size, weight, and faster and more flexible magnet movement [32].

### Amigo

The Amigo Remote Catheter System (Catheter Precision, Inc., Mount Olive, NJ, USA) was designed with the goal of providing a simple and less expensive solution for remote catheter manipulation in cardiac electrophysiology procedures [33]. The Amigo benefits from a handheld remote device as the control panel and compatibility with off-the-shelf ablation catheters. As a result of being designed specifically for cardiac electrophysiological treatments, this system has limited potential clinical application in PCI or PVI [34]. The safety and performance of the Amigo robotic system has been evaluated in a number of previous studies [35–38] that have been explained in further detail in Table 2.

### Other Current Endovascular Robotic Systems

Several other platforms are still under development, such as (1) Microbot Liberty (Microbot Medical Inc, MA, USA), (2) Endoways platform (Endoways, Or Yehuda, Israel), (3) Coral (Moray Medical, CA, USA), (4) DeepVessel AngioBot (Keya Medical, Beijing, PRC), (5) Shanghai Aopeng Medical’s platform (Shanghai Aopeng Medical Technology Co. Ltd, Shanghai, PRC), and (6) WeMed’s platform (WeMed, Beijing, PRC).

In parallel to the ongoing commercialisation efforts, a plethora of work has been reported in literature [6, 12]. Most recently, the ongoing research endeavours in developing magnetic resonance (MR) safe and MR conditional robotic platforms for MR-guided endovascular interventions [39]. Generally speaking, MRI offers unprecedented opportunities to combine diagnosis, therapy and early evaluation of therapy in a single endovascular intervention [40]. Researchers overcome the material constraints (i.e. inability to use ferromagnetic materials) of the highly magnetic MRI environment by replacing the commonly used electric motors with non-ferromagnetic ultrasonic motors [41] and plastic stepper motors [42, 43]. These versatile systems can help mitigate the challenges of performing manual MR-guided interventions by: (a) providing accessibility to patients inside the MRI bore (especially paediatric patients) and (b) reducing the physicians’ exposure to the uncomfortable acoustic noise, which may lead to hearing impairment [44]. Moreover, companies such as MaRVis Interventional GmbH (Krün, Germany), Nano4imaging (Düsseldorf, Germany) and EPFlex (Dettingen an der Erms, Germany) are complementing these advancements in robotics through their leading developments in the field of MR compatible instrumentation (i.e. MR safe and MR conditional guidewires) which could potentially pave the way for the broader adoption of MR-guidance in endovascular interventions.

## Robotic Non-vascular Systems

Interventional radiologists have successfully used various imaging modalities to guide their path to target and monitor their treatment outcome in a vast number of non-vascular interventions. The application of robotic systems in these CT- and MRI-guided procedures could aid in improving accuracy, precision and safety. In addition, it could reduce the high radiation exposure of CT scans to the physician and other healthcare staff. In this section, we will review some of the advancements in robotic CT- and MRI-guided systems in non-vascular IR procedures. A summary of the key characteristics of these robotic systems is listed in Table 3 and a summary of the clinical studies undertaken using these robots is found in Table 4.

**Table 3:** Summary of the main characteristics of robotic systems for non-vascular IR

Robotic system	Institute	Regulatory status	Imaging modalities	DOF of robot	Procedures
AcuBot	Hopkins/Georgetown, USA	FDA	Fluoroscopy, CT	6 DOF	Biopsy, drainage, tumour ablation, RFA, vertebroplasty
B-Rob II	ARC Seibersdorf Research, Austria	N/A	CT, US	7 DOF	Biopsies
iSYS1	Medizintechnik GmbH, Kitzbühel, Austria	CE mark, FDA	Fluoroscopy, CT, CBCT	4 DOF	Biopsy, catheter placement
Zerobot	Okayama University, Japan	N/A	CT	6 DOF	Biopsy, ablation, drainage
ROBIO EX	Perfint Healthcare Pvt. Ltd, Florence, OR, USA	CE mark	CT, PET-CT	5 DOF	Biopsy, ablation, drainage
INNOMOTION	Innomedic, Rheinsheim-Philippsburg, Germany	CE mark	CT, MRI	6 DOF	Biopsy, tumour ablation, drainage
EPIONE	Quantum Surgical, Montpellier, France	CE mark, FDA	CT	6 DOF	Tumour ablation

*DOF* degrees of freedom; *CT* computed tomography; *US* ultrasound; *CBCT* cone-beam computed tomography; *RFA* radiofrequency ablation

**Table 4:** Summary of the clinical studies for non-vascular robotic systems

Study	Robotic system	Type of study (# subjects)	Aim	Key findings
Cleary et al. [84]	AcuBot	RCT (20)	Comparing robotic versus manual nerve and facet block	9/10 correct placement of needle 1 subject required conversion to manual technique due to slippage of needle driver No peri-procedural complications
Minchev et al. [52]	iSYS1	Prospective, single arm (25)	Evaluation of robotic-assisted brain tumour biopsies and intracranial catheter placements	Median target error of 0.9 mm Average setup time: 11.8 min Average instrument positioning time: 4.9 min 100% diagnostic yield from biopsies Robotic assistance was not feasible in 1 patient due to an operator error All 5 shunts were appropriately placed
Vakharia et al. [53]	iSYS1	RCT (32)	Comparing robotic-guided versus manual approach in implantation of intracerebral electrodes	Median target point accuracy for manual was 1.16 mm versus 1.58 mm for iSYS1 (*p* = 0.004) Mean electrode implantation angular error for manual was 1.71° versus 2.13° for iSYS1 (*p* = 0.023)
Hiraki et al. [85]	Zerobot	Prospective, single arm (10)	Evaluation of robotic-assisted biopsies for lesions in the extremity or the trunk	100% of the introducer needle tip was inserted within < 10 mm from nearest lesion edge Mean CT fluoroscopy time: 29 s Mean operation time: 4 min 11 adverse outcomes (no robot-related issues)
Abdullah et al. [59]	Robio EX	Prospective, single arm (11)	Evaluation of robotic-assisted RFA of primary and secondary liver tumours	100% RFA completed 6 lesions required readjustment of needle No complications reported
Anzidei et al. [86]	Robio EX	RCT (100)	Comparing robotic CT-guided lung biopsy versus manual technique	Biopsies obtained in all cases No differences in precision of needle positioning, diagnostic yield from biopsies, and complications Average procedure time of robotic was 20.1 min versus 31.4 min for manual (*p* = 0.001) Average DLP of 324 mGy for robotic versus 541.2 mGy for manual (*p* < 0.05)
Kumar et al. [87]	Robio EX	Prospective, single arm (78)	Evaluation of PET-guided, robotic-assisted transgluteal prostate biopsy	Prostate cancer confirmed in 96% of patients 2 insufficient samples 9% post-procedure complications
de Baère et al. [60]	EPIONE	Prospective, single arm (21)	Evaluation of CT-guided percutaneous thermal ablation of liver tumours	One patient excluded due to protocol deviation Feasible thermal ablation in 95.7% of lesions No peri-procedural complications Two patients had died at 6 months follow up; cause of death unrelated to ablation procedure Local tumour control at 6 months was achieved in 83.3% of patients
Melzer et al. [66]	Innomotion	Prospective, single arm (16)	Evaluation of MR-guided robotic-assisted percutaneous facet joint treatment	100% procedures completed Some minor side effects: hyperhidrosis (*n* = 1), prolonged menstruation (*n* = 1) No major adverse events
Kettenbach et al. [88]	Innomotion	Prospective, single arm (12)	Evaluation of MR-guided biopsy, drainage, and tumour ablation in chest and abdominal cavities and retroperitoneum	100% procedures completed Medial overall operation time: 71 min Median puncture needle insertion length: 6.9 cm 100% diagnostic yield from biopsies 2/2 tumours fully necrosed 1/1 evacuation of pleural empyema No complications reported
Zangos et al. [89]	Innomotion	Prospective, single arm (20)	Evaluation of MR-guided, robotic-assisted transgluteal prostate biopsy	19/20 satisfactory biopsies Median deviation of needle tip to planned access was 0.9 mm Median procedure time: 39 min No procedural complications

*RCT* randomised control trial; *PAD* precision-aiming device; *CT* computed tomography; *RFA* radiofrequency ablation; *DLP* dose length product; *PET* positron emission tomography; *MRI* magnetic resonance imaging

### AcuBot

One of the first CT-compatible robotic systems was the AcuBot (URobotics Laboratory, The Johns Hopkins University, Georgetown, USA) [45]. The FDA-approved AcuBot was built on the previous PAKY-RCM robotic system and was improved with the addition of several new components including a passive S-arm and an XYZ Cartesian stage [45]. The robot has 6 DOF designed for decoupled positioning, orientation, and instrument insertion [45]. This robotic system has been tested in a cadaveric study for nerve and facet blocks, with an average placement accuracy of 1.44 ± 0.66 mm (mean ± SD) [46]. A recent gel phantom study compared the Acubot with a computer-assisted optical navigation system in the performance of percutaneous ablative targeting in gel phantom [47]. The mean translational offset from the predefined targets was 1.2 mm (range 0.39–2.82 mm) for the AcuBot system and 5.8 mm (range 1.8–11.9 mm) for the navigation system. The AcuBot was also faster to reach target with an average of 37 s (range 15–75), compared to 108 s (range 45–315) for the navigation system [47].

### B-Rob II

The B-Rob II robotic system (Austrian Research Group ARC, Seibersdorf Research, Austria), the successor of the B-Rob I, has 7 DOF and has been designed for both CT- and Ultrasound (US)-guided biopsy sampling. This second-generation robot was designed with the aim of creating a flexible setup design that was better suited for clinical practice, with easier integration with other systems while reducing technical complexity and costs. The accuracy of robotic needle placement of the B-Rob II system was evaluated using a gelatin phantom with 21 biopsies performed [48]. The average needle placement accuracy was 1.8 ± 1.1 mm (mean ± SD), and the average procedure time was 2 min 21 s [48]. More recently, this robotic system was used to assist post-mortem CT-guided biopsies for foetus and infants; however, it provided limited additional diagnostic value [49]. The authors explained that biopsy sampling failure mostly involved organs with reduced soft tissue contrast on CT, such as the spleen, and that evaluation of these organs in foetuses with low abdominal and subcutaneous fat is generally difficult.

### iSYS1

The iSYS1 robot system (iSYS Medizintechnik GmbH, Kitzbuehel, Austria) is the successor of B-Rob II system. The iSYS1 robot received its CE mark and FDA approval in 2013 and 2014, respectively, and has since been used in pre-clinical and clinical settings [50–53]. The robot is compatible with cone beam CT (CBCT) as well as CT/fluoroscopy. The robot has a four axial robotic positioning unit, which consists of a 2 DOF translational workspace measuring 40 × 40 mm and another 2 DOF angulation of ± 32 degree of the needle [54, 55]. In a phantom study, the iSYS1 robot successfully performed 40 needle target punctures, with 20 targets in single and 20 in double oblique trajectories. Overall, the mean length of the target path was 8.5 cm (range 4.2–13.5 cm) from the phantom surface. For all procedures, the average duration was 3 min 59 s with an overall needle tip deviation of 1.1 mm (range 0–4.5 mm) from the predefined path [50]. Another study utilised the iSYS1 robotic system for CT-guided punctures of targets placed in a torso phantom [51]. The mean difference between the depth of the planned needle trajectories with the actual needle placements was 1.3 ± 1.2 mm. The authors also reported the mean Euclidean distance between the target and the actual needle tip as 2.3 ± 0.9 mm, and concluded that accurate needle placement near small targets was feasible with the iSYS1 robotic system [51].

### Zerobot

The Zerobot (designed by Okayama University; manufactured by Medicalnet Okayama) is another remote-controlled robot designed for CT-guided procedures requiring needle insertion, such as ablation, biopsy, and drainage [56]. The Zerobot has an operation interface that can manipulate the robot with 6 DOF. Following an experiment through which the robot yielded accurate and safe results in phantom and animal experiments [57], the robot was used in needle orientation and insertion under CT guidance using four different ablation needle types in six swine, aiming for targets in the liver, kidney, lung, and hip muscle [58]. It was found that the overall mean accuracy of all needles for all targets was 2.8 ± 1.0 mm (mean ± SD).

### Robio EX

The Robio EX (Perfint Healthcare Pvt. Ltd, Florence, USA) is another CE-marked robotic system that is compatible with CT and positron emission tomography (PET)-CT. The Robio EX’s robotic arm has 5 DOF movement with two linear motions for positioning of the guide and two angular motions to modify the needle to the appropriate angular entry [59]. This robotic system was designed for thoracic and abdominal interventions, including biopsy, drainage, and tumour ablation. It also includes a breath hold management system in order to secure targets that may move due to respiratory effort. One main disadvantage of the Robio EX is that it is situated on its stand which fixed to the floor, and as such the needle must be decoupled every time the CT table is moved.

### EPIONE

The EPIONE robotic system (Quantum Surgical, Montpellier, France), both CE marked, and FDA cleared, is another robotic system used in CT-guided percutaneous needle insertion. The EPIONE robotic system has 6 DOF and is comprised of five components: the mobile arm (1) which has attached to it the needle guide (2), an infra-red camera (3) acting as the navigation cart, a workstation (4), and patient reference (5) which is adhesively attached to the patient’s skin and allows tracking of patient’s respiratory cycle [60]. This robotic system has been safely used in CT-guided percutaneous needle placement for targeting of previously implanted fiducials in the liver of ten swine [61]. Similarly, the robot was used in CT-guided percutaneous needle insertion targeting a total of eight fiducial targets placed in the kidneys of two swine. All needle insertions successfully reached the target on the first attempt with no need for readjustment; however, there were two subcapsular haematomas which did not progress to retroperitoneal effusions [62]. In a recent prospective study, the EPIONE robotic system was used for robotic-assisted thermal ablation of liver tumours [60].

### INNOMOTION

MRI has slowly become a popular choice of imaging modality in interventional procedures mainly due to the excellent soft tissue contrast resolution, the lack of ionising radiation, and the ability for multimodality sensing such as blood flow, motion, deformation, strain, and temperature [63]. However, as previously mentioned, it has major disadvantages including cost, the limited bore space, and the constraints on compatible instruments [64]. One robotic system that is both CT- and MR-compatible is INNOMOTION (Innomedic, Herxheim, FZK Karlsruhe, TH Gelsenkirchen, Germany). The second generation INNOMOTION robotic arm has 6 DOF with an additional passive rotation DOF for prepositioning and was developed with the main goal of accurate instrument positioning inside the magnet [65]. This robotic system involves a robotic arm attached to a ring which is subsequently mounted onto the patient table. The target precision of the robotic system under MR guidance was tested in porcine kidney embedded in gelatin phantom [66]. Based on the results, INNOMOTION received a CE mark for percutaneous interventions.

### Other Current Non-vascular MRI Robotic Systems

In addition to the MRI-guided robotic systems mentioned here, there are numerous other robotic systems that have or are currently undergoing further testing in different interventions, such as for prostate biopsies [67, 68], breast biopsy [69–71], lumbar spine injections [72], shoulder arthrography [73, 74], and neuroablation [75].

## Discussion and Conclusion

**Fig. 1:**
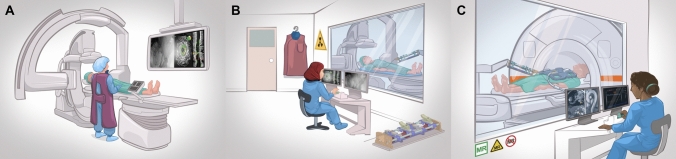
**A** Current, routine IR procedures during which the physician is exposed to ionising X-ray radiations, which may lead to a sizeable risk of cancer. The physician is also wearing a heavy lead apron, which makes the physician more prone to orthopaedic injuries. **B** Physician is remotely operating a robotic platform. The physician is no longer exposed to ionising radiations and no longer needs to wear the lead apron. The robotic platform may also improve the precision, stability, and comfort of endovascular procedures. **C** Physician is remotely operating a robotic platform inside an MRI scanner instead of using X-ray guidance. MRI does not have any ionising radiation, which makes it a safe option for patients, especially the paediatric population. MRI can also provide better visualisation of soft tissue and offers unparalleled 3D evaluations of pathology and function across the body

Recent advances in robotic platforms and technologies have resulted in improvements in robotic-assisted endovascular and non-vascular procedures. Robotic systems in IR can address one of the few downsides of this field, which is the exposure to ionising radiation to both patients and healthcare staff (Fig. 1). In addition, other potential benefits that have been claimed using robotic systems in IR include increased accuracy and precision, reduced operation time, and reduced numbers of readjustments needed to reach target. Ultimately, with further advancements in remotely controlled robotic systems, robotic-assisted IR may lead to improved access to healthcare, especially in rural areas. In combination with surgical simulators, robotic systems can be used as a potential training tool in the future that will allow highly accurate training scenarios with minimised radiation exposure. Similarly, the use of robotic systems may lead to minimisation of user-variability in future interventions. However, there are still a number of drawbacks that need to be addressed to allow widespread adoption of this technology in the field of IR. Some of these limitations include the high cost of these robots, the inability to integrate some robotic systems with other surgical devices and/or instruments, the interference to workflow in the IR suite, and the lack of haptic feedback. The application of artificial intelligence (AI) to robotic surgery has shown some promise in improving surgical parameters, such as improved haptic feedback systems and surgical guidance, as well as better prediction of operative time and post-op outcomes [76]. Thereby, the integration of AI with robotic systems in IR may address some of the current pitfalls of these systems.

In conclusion, robotic guided interventions are continuously developing with established safety records and promising efficacy prospects. While the balance between efficacy and cost implications needs to be considered, interventional radiologists should be continuously engaged and lead the robotic development in the field of vascular and oncology interventions to maximise the benefits to patients and operators.

## References

[1] Himpens J, Leman G, Cadiere GB (1998). Telesurgical laparoscopic cholecystectomy. Surg Endosc.

[2] Jacofsky DMD, Allen MDO (2016). Robotics in arthroplasty: a comprehensive review. J Arthroplasty.

[3] Zhao Y (2022). Remote vascular interventional surgery robotics: a literature review. Quant Imaging Med Surg.

[4] Riga C (2009). Initial clinical application of a robotically steerable catheter system in endovascular aneurysm repair. J Endovasc Ther.

[5] Beaman CB (2021). A review of robotic interventional neuroradiology. Am J Neuroradiol AJNR.

[6] Antoniou GAMD (2011). Clinical applications of robotic technology in vascular and endovascular surgery. J Vasc Surg.

[7] Rafii-Tari H, Payne CJ, Yang GZ (2014). Current and emerging robot-assisted endovascular catheterization technologies: a review. Ann Biomed Eng.

[8] Lumsden AB, Bismuth J (2018). Current status of endovascular catheter robotics. J Cardiovasc Surg (Torino).

[9] Rolls A, Riga C (2018). Endovascular robotics. Ann R Coll Surg Engl.

[10] Chi W. Context-aware learning for robot-assisted endovascular catheterization. Department of Computing, Imperial College London;2020.

[11] Legeza P (2020). Current utilization and future directions of robotic-assisted endovascular surgery. Expert Rev Med Devices.

[12] Gunduz S, Albadawi H, Oklu R (2021). Robotic devices for minimally invasive endovascular interventions: a new dawn for interventional radiology. Adv Intell Syst.

[13] Püschel A, Schafmayer C, Groß J (2022). Robot-assisted techniques in vascular and endovascular surgery. Langenbecks Arch Surg.

[14] Cruddas L, Martin G, Riga C (2021). Robotic endovascular surgery: current and future practice. Semin Vasc Surg.

[15] Shurrab M (2014). Robotics in invasive cardiac electrophysiology. Expert Rev Med Devices.

[16] Carrell T (2012). Use of a remotely steerable “robotic” catheter in a branched endovascular aortic graft. J Vasc Surg.

[17] Cercenelli L, Bortolani B, Marcelli E (2017). CathROB: a highly compact and versatile remote catheter navigation system. Appl Bionics Biomech.

[18] Riga CV (2013). Robot-assisted fenestrated endovascular aneurysm repair (FEVAR) using the Magellan system. J Vasc Interv Radiol.

[19] Rolls AE (2014). Robot-assisted uterine artery embolization: a first-in-woman safety evaluation of the Magellan System. J Vasc Interv Radiol.

[20] Jones B (2021). Robot-assisted carotid artery stenting: a safety and feasibility study. Cardiovasc Interv Radiol.

[21] Rafii-Tari H (2016). Reducing contact forces in the arch and supra-aortic vessels using the Magellan robot. J Vasc Surg.

[22] Perera AH (2017). Robotic arch catheter placement reduces cerebral embolization during thoracic endovascular aortic repair (TEVAR). Eur J Vasc Endovasc Surg.

[23] Cheung S (2020). Comparison of manual versus robot-assisted contralateral gate cannulation in patients undergoing endovascular aneurysm repair. Int J Comput Assist Radiol Surg.

[24] Riga CV (2012). Tortuous iliac systems—a significant burden to conventional cannulation in the visceral segment: Is there a role for robotic catheter technology?. J Vasc Interv Radiol.

[25] Al Nooryani A, Aboushokka W (2018). Rotate-on-retract procedural automation for robotic-assisted percutaneous coronary intervention: first clinical experience. Case Rep Cardiol.

[26] Caputo R, Lesser A, Simons A (2015). CRT-313 feasibility of robotic percutaneous renal artery revascularization. JACC Cardiovasc Interv.

[27] Mendes Pereira V (2020). First-in-human, robotic-assisted neuroendovascular intervention. J NeuroInterv Surg.

[28] Piotin M (2022). P15 Evaluation of effectiveness and safety of the CorPath® GRX System in endovascular embolization procedures of cerebral aneurysms. J NeuroInterv Surg.

[29] CorPath® GRX Neuro Study. Available from: https://ClinicalTrials.gov/show/NCT04236856.

[30] Rueda MA, Riga CT, Hamady MS (2018). Robotics in interventional radiology: past, present, and future. Arab J Interv Radiol.

[31] Eric D, et al. Evaluation of the R-one robotic system for percutaneous coronary intervention: the R-EVOLUTION study. EuroIntervention;2023. 10.4244/EIJ-D-22-00642 PMC10068861 36602883

[32] Hwang J, Kim J-Y, Choi H (2020). A review of magnetic actuation systems and magnetically actuated guidewire- and catheter-based microrobots for vascular interventions. Intel Serv Robot.

[33] Shaikh ZA, Eilenberg MF, Cohen TJ (2017). The Amigo™ remote catheter system: from concept to bedside. J Innov Card Rhythm Manag.

[34] Crinnion W (2022). Robotics in neurointerventional surgery: a systematic review of the literature. J NeuroInterv Surg.

[35] Khan EM (2013). First experience with a novel robotic remote catheter system: Amigo™ mapping trial. J Interv Card Electrophysiol.

[36] Datino T (2014). Comparison of the safety and feasibility of arrhythmia ablation using the Amigo Robotic Remote Catheter System versus manual ablation. Am J Cardiol.

[37] López-Gil M (2014). Cavo-tricuspid isthmus radiofrequency ablation using a novel remote navigation catheter system in patients with typical atrial flutter. Europace.

[38] Wutzler A (2014). Robotic ablation of atrial fibrillation with a new remote catheter system. J Interv Card Electrophysiol.

[39] Abdelaziz MEMK (2021). X-ray to MR: the progress of flexible instruments for endovascular navigation. Prog Biomed Eng.

[40] Bock M, Wacker FK (2008). MR-guided intravascular interventions: Techniques and applications. J Magn Reson Imaging.

[41] Tavallaei MA (2013). A magnetic-resonance-imaging-compatible remote catheter navigation system. IEEE Trans Biomed Eng.

[42] Abdelaziz MEMK (2019). Toward a versatile robotic platform for fluoroscopy and MRI-guided endovascular interventions: a pre-clinical study. IEEE/RSJ Int Conf Intell Robots Syst (IROS).

[43] Kundrat D (2021). An MR-safe endovascular robotic platform: design, control, and ex-vivo evaluation. IEEE Trans Biomed Eng.

[44] McJury M, Shellock FG (2000). Auditory noise associated with MR procedures: a review. J Magn Reson Imaging.

[45] Stoianovici D (2003). AcuBot: a robot for radiological interventions. IEEE Trans Robot Autom.

[46] Cleary K (2002). Robotically assisted nerve and facet blocks: a cadaveric study. Acad Radiol.

[47] Pollock R (2010). Prospects in percutaneous ablative targeting: comparison of a computer-assisted navigation system and the AcuBot Robotic System. J Endourol.

[48] Martinez RM (2014). CT-guided, minimally invasive, postmortem needle biopsy using the B-Rob II needle-positioning robot. J Forensic Sci.

[49] Rüegger CM (2022). Post-mortem magnetic resonance imaging with computed tomography-guided biopsy for foetuses and infants: a prospective, multicentre, cross-sectional study. BMC Pediatr.

[50] Schulz B (2013). Accuracy and speed of robotic assisted needle interventions using a modern cone beam computed tomography intervention suite: a phantom study. Eur Radiol.

[51] Kettenbach J (2014). A robotic needle-positioning and guidance system for CT-guided puncture: ex vivo results. Minim Invasive Ther Allied Technol.

[52] Minchev G (2017). A novel miniature robotic guidance device for stereotactic neurosurgical interventions: preliminary experience with the iSYS1 robot. J Neurosurg.

[53] Vakharia VN (2021). Comparison of robotic and manual implantation of intracerebral electrodes: a single-centre, single-blinded, randomised controlled trial. Sci Rep.

[54] Minchev G, et al. Application of the ISYS1 robotic device for stereotactic neurosurgical interventions: a preclinical phantom trial. In CURAC. 2013.

[55] Kettenbach J, Kronreif G (2015). Robotic systems for percutaneous needle-guided interventions. Minim Invasive Ther Allied Technol.

[56] Hiraki T (2018). Zerobot®: a remote-controlled robot for needle insertion in CT-guided Interventional Radiology Developed at Okayama University. Acta Med Okayama.

[57] Hiraki T (2017). Robotically driven CT-guided needle insertion: preliminary results in phantom and animal experiments. Radiology.

[58] Hiraki T (2018). Robotic insertion of various ablation needles under computed tomography guidance: accuracy in animal experiments. Eur J Radiol.

[59] Abdullah BJ (2014). Robot-assisted radiofrequency ablation of primary and secondary liver tumours: early experience. Eur Radiol.

[60] de Baère T (2022). Evaluation of a new CT-guided robotic system for percutaneous needle insertion for thermal ablation of liver tumors: a prospective pilot study. Cardiovasc Intervent Radiol.

[61] Guiu B (2021). Feasibility, safety and accuracy of a CT-guided robotic assistance for percutaneous needle placement in a swine liver model. Sci Rep.

[62] de Baere T (2022). Robotic assistance for percutaneous needle insertion in the kidney: preclinical proof on a swine animal model. Eur Radiol Exp.

[63] Su H (2022). State of the art and future opportunities in MRI-guided robot-assisted surgery and interventions. Proc IEEE Inst Electr Electron Eng.

[64] Kassamali RH, Ladak B (2015). The role of robotics in interventional radiology: current status. Quant Imaging Med Surg.

[65] Cleary K (2006). Interventional robotic systems: applications and technology state-of-the-art. Minim Invasive Ther Allied Technol.

[66] Melzer A (2008). INNOMOTION for percutaneous image-guided interventions: principles and evaluation of this MR- and CT-compatible robotic system. IEEE Eng Med Biol Mag.

[67] Stoianovici D (2008). MRI-compatible Pneumatic Robot (MRBot) for prostate brachytherapy: preclinical assessment of accuracy and execution of dosimetric plans. Int J Radiat Oncol Biol Phys.

[68] Stoianovici D (2017). MR safe robot, FDA clearance, safety and feasibility prostate biopsy clinical trial. IEEE ASME Trans Mechatron.

[69] Yang B (2014). Design, development, and evaluation of a master-slave surgical system for breast biopsy under continuous MRI. Int J Robot Res.

[70] Chan KG, Fielding T, Anvari M (2016). An image-guided automated robot for MRI breast biopsy. Int J Med Robot Comput Assist Surg.

[71] Groenhuis V, et al. Design and characterization of Stormram 4: an MRI-compatible robotic system for breast biopsy. In 2017 IEEE/RSJ international conference on intelligent robots and systems (IROS);2017.

[72] Li G (2020). Fully actuated body-mounted robotic system for MRI-guided lower back pain injections: initial phantom and cadaver studies. IEEE Robot Autom Lett.

[73] Patel NA, et al. Robotic system for MRI-guided shoulder arthrography: accuracy evaluation. In 2018 International symposium on medical robotics (ISMR;2018.

[74] Patel N (2019). Preclinical evaluation of an integrated robotic system for magnetic resonance imaging guided shoulder arthrography. J Med Imaging (Bellingham).

[75] Patel NA (2020). An integrated robotic system for MRI-guided neuroablation: preclinical evaluation. IEEE Trans Biomed Eng.

[76] Andras I (2020). Artificial intelligence and robotics: a combination that is changing the operating room. World J Urol.

[77] Smitson CC (2018). Safety and feasibility of a novel, second-generation robotic-assisted system for percutaneous coronary intervention: first-in-human report. J Invasive Cardiol.

[78] Swaminathan RV, Rao SV (2018). Robotic-assisted transradial diagnostic coronary angiography. Catheter Cardiovasc Interv.

[79] Hirai T (2020). A case of robotic assisted percutaneous coronary intervention of the left main coronary artery in a patient with very late baffle stenosis after surgical correction of anomalous left coronary artery from the pulmonary artery. Catheter Cardiovasc Interv.

[80] Da Costa A (2017). Substantial superiority of Niobe ES over Niobe II system in remote-controlled magnetic pulmonary vein isolation. Int J Cardiol.

[81] Yuan S (2017). Long-term outcomes of the current remote magnetic catheter navigation technique for ablation of atrial fibrillation. Scand Cardiovasc J.

[82] Kataria V (2017). Remote magnetic versus manual navigation for radiofrequency ablation of paroxysmal atrial fibrillation: long-term, controlled data in a large cohort. Biomed Res Int.

[83] Luo Q (2022). Utilization of steerable sheath improves the efficiency of atrial fibrillation ablation guided by robotic magnetic navigation compared with fixed-curve sheath. Clin Cardiol.

[84] Cleary K (2005). Precision placement of instruments for minimally invasive procedures using a "needle driver" robot. Int J Med Robot.

[85] Hiraki T (2020). Robotic needle insertion during computed tomography fluoroscopy-guided biopsy: prospective first-in-human feasibility trial. Eur Radiol.

[86] Anzidei M (2015). Preliminary clinical experience with a dedicated interventional robotic system for CT-guided biopsies of lung lesions: a comparison with the conventional manual technique. Eur Radiol.

[87] Kumar R (2022). Safety and Diagnostic Yield of (68)Ga Prostate-specific Membrane Antigen PET/CT-guided Robotic-assisted Transgluteal Prostatic Biopsy. Radiology.

[88] Kettenbach J (2008). Abstract No. 157: pneumatically driven robotic system for MR-guided biopsie, drainage and tumorablation: first clinical experiences. J Vasc Interv Radiol.

[89] Zangos S (2011). MR-compatible assistance system for biopsy in a high-field-strength system: initial results in patients with suspicious prostate lesions. Radiology.

